# Personal Protection Equipment and Infection Control Procedures among Health Workers during the COVID-19 Pandemic

**DOI:** 10.3390/healthcare10050944

**Published:** 2022-05-19

**Authors:** Daniela Carmagnola, Marilisa Toma, Dolaji Henin, Mariachiara Perrotta, Gaia Pellegrini, Claudia Dellavia

**Affiliations:** Department of Biomedical, Surgical and Dental Sciences, Università degli Studi di Milano, Via Mangiagalli 31, 20133 Milano, Italy; marilisa.toma@unimi.it (M.T.); dolaji.henin@unimi.it (D.H.); mariachiara.perrotta1997@gmail.com (M.P.); gaia.pellegrini@unimi.it (G.P.); claudia.dellavia@unimi.it (C.D.)

**Keywords:** COVID-19, dental care professionals, general practitioners, PPE, exposure-prone

## Abstract

Health workers have been particularly affected by the COVID-19 pandemic, but it is unclear which healthcare professions are more exposed. We search for information that can help identify health workers who are employed in exposure-prone professions and who, therefore, routinely wear and adopt strict infection control equipment and measures from other colleagues. Our purpose is to test the hypothesis that health professionals historically considered less exposure-prone are affected more severely by COVID-19. Taking dentists as an example of exposure-prone healthcare professionals, this study aims to analyze data on COVID-19-related deaths reported by the Italian board of doctors and dentists’ database to evaluate the number of COVID-19-related deaths of doctors and dentists in Italy from the beginning of the pandemic to 31 December 2022. As of 31 December 2021, out of 364 deaths, 38 were dentists, and of the remaining 326 doctors, 140 were general practitioners (GPs). The percentage of deaths among dentists, total doctors and GPs results in 0.06%, 0.09% and 0.33%, respectively, for the whole sample. Excluding subjects over 70 years of age, the corresponding values are 0.05%, 0.06% and 0.25%. Most of the deaths occurred in Lombardia, and the geographical distribution overlaps the trend of the corresponding general Italian population. Considering the outcome of “death”, dentists, despite being at high risk, are not particularly affected by COVID-19.

## 1. Introduction

The coronavirus disease, COVID-19, is caused by severe acute respiratory syndrome coronavirus 2 (SARS-CoV-2), an RNA virus first identified in Wuhan, China, in December 2019 [1]. Between February and April 2020, Italy and, in particular, its northern regions, experienced one of the largest clusters and case fatality rates of COVID-19 worldwide [2]. Such an event led to a total lockdown for weeks. During the first and following waves, depending on epidemiological data and the clinical characteristics of the various SARS-CoV-2 variants, a range of different non-pharmaceutical interventions (NPIs) was introduced, including limitations of personal movements and restrictions concerning business and activities, to try to control disease transmission. Similar patterns, both concerning COVID-19 transmission trends as well as the introduction of NPIs, occurred in different countries worldwide [3]. COVID-19 was defined as a pandemic by the World Health Organization (WHO) on 11 March 2020 [4].

Medical care was initially restricted to those with severe COVID-19-related symptoms or to emergencies, and most healthcare staff were deployed to COVID-19 facilities. An immediate urgent concern emerged regarding the safety of healthcare workers, and the WHO rapidly issued recommendations on the use of appropriate personal protection equipment (PPE). A medical mask, eye or facial protection, and a clean, non-sterile, long-sleeved gown and gloves were recommended during routine care. In the case of aerosol-generating procedures, such as tracheal intubation, non-invasive ventilation, tracheotomy, cardiopulmonary resuscitation, manual ventilation before intubation and bronchoscopy, which have been associated with an increased risk of transmission of the coronavirus, further measures are required, such as the use of a certified N95 or FFP2/3 respirator [5]. Nevertheless, a shortage of PPE was reported in many countries in the first weeks of the pandemic [6,7].

Such recommendations were issued and stressed due to the emergency, despite that, in principle, all patients should be considered potentially at risk to transmit infections. In addition, the importance of PPE and disinfection procedures to prevent cross infections in any medical setting was widely recognized before COVID-19. Nevertheless, not all healthcare personnel always found it easy to comply with them, because of, among other reasons, “unclear protocols, inconsistent training, climate challenges, and cultural sensitivities” [8,9]. Furthermore, among healthcare professions, some have always been considered more exposure-prone than others concerning infections, a distinction that may have misled some health workers to a false perception of safety.

Despite efforts to protect health workers, the severity and contagiousness of SARS-CoV-2 infections, elevated numbers of hospitalizations of very compromised patients, some inexperience and an initial shortage of PPE [10,11] are all factors that most likely contributed to the high rate of disease and deaths that occurred among healthcare professionals. As of 22 June 2020, therefore, within the first wave, according to the Italian National Health Institute [12], in Italy 29,282 healthcare professionals had been infected by COVID-19, which was about 12% of the overall officially infected Italian population (239.627 known cases), a proportion increasing to 20% in the Lombardy region [2]. As of February 2022, out of a total of 11,465,500 official COVID-19 cases in Italy since the start of the pandemic, 231,227 concerned health workers. With time, the proportion decreased to 2% [13]. No further details have been reported regarding which category these health workers belonged to. According to the WHO, about 180,000 heath workers may have died worldwide of COVID-19 between January 2020 and May 2021. The corresponding number for Italy was estimated to be 3.970 cases [14]. Moreover, in this case, no distinction concerning the type of specialization or task was provided. On 20 February 2022, during the celebrations of the Italian “national day for health workers”, the government reported that, since the beginning of the pandemic, about 500 health workers had died. A total of 90 were nurses, 3 were obstetricians, 32 were pharmacists and 370 included doctors and dentists. Of those 370 subjects, more than 200 were general practitioners (GPs) and public and private territorial practitioners, and about 30 were dentists.

Dentistry is considered particularly exposure-prone, as dental care procedures deal with body fluids and are aerosol-generating [15,16,17,18]. For exposure-prone professions [19], regardless of a patient’s history, the use of PPE and the adoption of infection control measures have, therefore, been routinary since well before COVID-19 [15,16,19]. A few weeks after the February 2020 COVID-19 outbreak in which they worked with “routine” PPE, public and private dental care professionals, concomitantly with the introduction of stay-at-home and other regulatory measures, stopped or limited their activity in Italy as well as in other countries [20,21,22].

The numbers and categories of the dead health professionals acknowledged by the Italian government during a press conference led us to wonder whether it is possible to better characterize which health workers had been more affected by COVID-19. In particular, we searched for information that could help identify those employed in exposure-prone professions, therefore hypothetically identifying those who routinely wear and adopt strict infection control equipment and measures from other colleagues. Our purpose was to test the hypothesis that health professionals who are historically considered less exposure-prone and therefore less acquainted to a routinary use of PPE may be affected more severely by COVID-19, and vice versa. Therefore, we analyzed data from the FNOMCEO (Federazione Nazionale degli Ordini dei Medici Chirurghi e degli Odontoiatri) database.

Affiliation to FNOMCEO, that is the Italian board of doctors and dentists, and in particular to its medical register for doctors and a separate dental register for dentists is compulsory in order to practice in Italy. FNOMCEO has updated the number of COVID-19-related deaths among its associates daily since the start of the pandemic [23]. Taking dentists as an example of exposure-prone healthcare professionals, the aim of this study is to analyze data from the FNOMCEO database to evaluate the number of COVID-19-related deaths of doctors and dentists in Italy from the beginning of the pandemic to 31 December 2022.

## 2. Materials and Methods

The FNOMCEO website provides an open section [24] that allows one to search for information concerning doctors and dentists (demographic search). By typing the name of a professional, the site displays the date and place of birth, affiliation province, university and year of degree (medical or dental school), university and year of any specialties and whether they are registered as a doctor, dentist or both.

As mentioned, since the start of the pandemic, a dedicated page of the FNOMCEO website regularly acknowledges the names, date of death and professions of its associates reported to have died of COVID-19 [23]. In our analysis, we extrapolated all available professional, demographic and geographical data by crossing the names on the list with the board’s public files. Further available information was gathered by searching on the internet, one by one, for the names of dead doctors and dentists, including data from the hospitals, practices or facilities they had worked at. By searching for their names, we observed that the deaths of health staff, especially general practitioners, were very often accompanied by a large echo on local newspapers, in which information was sometimes usefully integrated.

According to FNOMCEO in March 2021, Italy counted 63,596 dentists, 396,288 doctors and 26,169 double memberships, for a total of 459,884 members. GPs were about 42,000 (42,428 in 2020) [25].

Using data from the Italian Statistics Agency (ISTAT) [26] concerning COVID-19 mortality rates in the Italian population, we calculated standardized mortality rates for doctors, dentists and GPs.

## 3. Results

As of 31 December 2021, the FNOMCEO dedicated page reported the COVID-19-related death of 364 doctors and dentists in Italy. A total of 17 were women, and the average general age was 69 years (Table 1). For most of them, a public personal record was available on the FNOMCEO website. We extrapolated and reported data, when verified, concerning the overall number, GP, dentists and remaining subjects (including doubtful files). A total of 38 were dentists, either exclusively (16 cases) or with both a medical and dental license (22 cases, 11 of whom worked at the same time as dentists and GPs). Of the remaining 326 doctors, 140 (38% of the whole sample population) were GPs, though 71 had officially retired, and some others, being over 70, were most likely former GPs currently practicing privately. Therefore, in Table 2, we report the age distribution, excluding subjects >70 years of age as well as the 24 doctors/dentists whose age was unknown. The results reported both in Table 1 and Table 2 show a similar average age among groups, and the outcome without the population of those whose age was >70 resulted in a lower average age of 3 to 7 years. The percentage of deaths among dentists, total doctors and GPs resulted in 0.06%, 0.09% and 0.33%, respectively, for the whole sample. Excluding subjects over 70 years of age, the corresponding values were 0.05%, 0.06% and 0.25%.

The standardized mortality rates are reported in Table 3. They show that the whole sample of doctors had a similar mortality rate to the general Italian population, whereas dentists showed a slight increase in the age group 55–64. GPs showed the highest values, starting from the same age group (55–64) but increasing dramatically with increasing age, showing a peak in the >75 age group.

**Figure 1:**
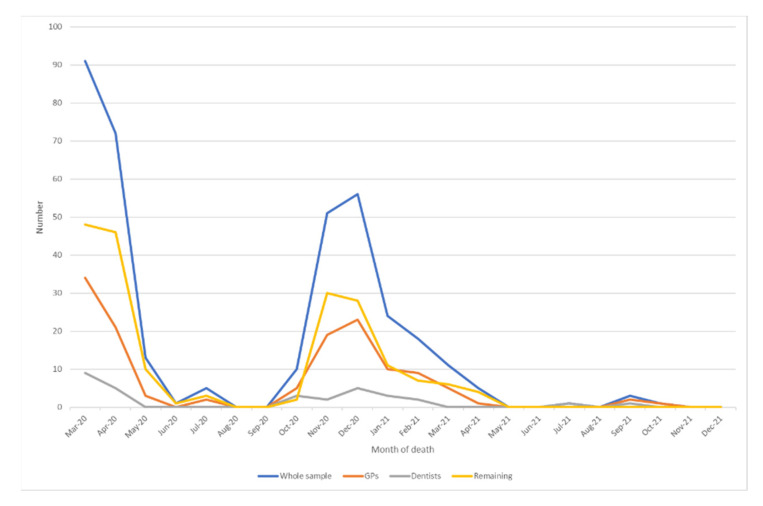
Time distribution of the cases (deaths) of the whole sample, GPs, dentists and remaining doctors.

The first death was reported on 11 March, and the distribution of deaths per group in time is shown in Figure 1. A peak was observed between March and April 2020, followed by a lower second one between November and December of the same year (therefore corresponding to the first and second Italian waves). The lines display similar trends, with a possible flatter course for dentists during the second wave.

The geographic distribution of the deaths is reported in Table 4, which groups Northern, Central and Southern Italian regions. A total of 60% of the deaths occurred in Northern Italy, and, more specifically, 33% occurred in Lombardia, with Bergamo (29 cases), Milano (21 cases) and Brescia (16 cases) being the most affected provinces. A total of 46 of the dead GPs worked in Lombardia.

## 4. Discussion

In our study, by retrospectively analyzing data retrieved from the Italian board of doctors and dentists and using death as an outcome, we observed that doctors and dentists were similarly affected by CPVOD-19, concerning both numbers and temporal trends. Among the medical categories that can be identified, the number of deaths among GPs was three times higher than the number of deaths among dentists (0.25 to 0.33% for GPs compared to <0.01% for other doctors and dentists). The standardized mortality rates seem to confirm the same trend, even though the highest values of the GPs were observed for the >75 age group, when most of them had possibly retired. However, many doctors tended to continue their activity privately. Furthermore, during the pandemic in Italy, retired doctors were recruited to support the health system.

The dataset we used has obvious limitations. Firstly, the list of COVID-19-related deaths does not include any information on subjects’ medical history and comorbidities, nor does it include whether they may have been exposed at their workplace by patients or in the community. Data were not available concerning the SARS-CoV-2 serology or PPE supply of each single health worker. Furthermore, we could only discriminate between dentists, GPs and remaining doctors, and we were quite aware of the fact that the remaining doctors category included many exposure-prone health professionals from various specialties. Moreover, we analyzed the “death” outcome, and we cannot be sure that it reflects infection and disease severity trends. Moreover, the same bias applies for the whole sample we considered as well as for the documented initial PPE shortage, especially concerning surgical and FFP2/3 masks, gowns and disinfectants. Furthermore, information on the board’s website is the only available source we found that allowed for the identification of a specific high-risk group of professionals, and our analysis seems to be in agreement with national and international data [20,22] reporting that COVID-19 did not impact dental professionals more than health workers of other categories. As we lacked sufficient information to further differentiate among doctors, in our study we did not aim to compare among categories, but rather we used dentists to represent an identifiable example of exposure-prone professionals.

After the outbreak of the disease and during the pandemic, as mentioned, medical care was restricted to those with severe COVID-19-related symptoms or to emergencies, and most healthcare staff were deployed to COVID-19 facilities. The same auto-regulatory approach was adopted by dentists. With time, however, activities slowly re-opened or returned to standard levels of occupation, adapting PPE choice and procedures to guidelines and recommendations released over time [11,27,28,29,30]. Data from a survey in Lombardia confirm that almost half of dentists continued to work to some extent after the outbreak of the disease [22]. Nevertheless, according to recent data, dentists were not particularly affected by COVID-19. A multicenter international study performed in 36 countries gathering information on the effects of COVID-19 on dental practice reported that rates of COVID-19 for dental professionals were not significantly different to those regarding the general population in each country [20].

In dentistry, routine PPE and procedures include wearing surgical masks, eyes/face protection, gloves and dedicated clothes; cleaning all surfaces with disinfectants between patients; using sterile instruments for each patient; and working with dedicated assisting/administrative personnel [16]. As a matter of fact, documented cross infections in the dental setting are extremely rare [31], supporting the assumption that adherence to strict PPE and infection control protocols is effective in preventing infection transmission and keeping caregivers and patients reciprocally safe.

Among the explanations for such an outcome, both concerning more “traditional” infections as well as for COVID-19, the role of PPE and strict infection control protocols has been advocated [20,22]. Proving that the use of PPE and a strict adherence to infection control protocols have played a significant role in the prevention of COVID-19 transmission in the dental setting is beyond the scope and possibilities of this study. We can, nevertheless, speculate that the routine use of PPE may have been sufficient to not put dental professionals at higher risk compared to other colleagues. A Cochrane review [32] on the use of PPE by healthcare staff has substantially concluded that frequent, inappropriate use of PPE as well as donning and doffing mistakes can lead to augmented contamination, and that guidance and information may reduce errors. A study from 2016, at the time of the Ebola outbreak, concluded how both access to PPE as well as “adherence to appropriate PPE use” are challenges due to inadequate education on its usage, technical difficulties and tolerability of PPE in the workplace [33]. A recent Brazilian study evaluating the rate of occupational biological accidents among physicians, dentists and nurses in a university setting reported that “The levels of knowledge and adherence to standard procedures were good, with the best found in dentists and dental students” [34]. A study from Hong Kong [35] investigating the risk of nosocomial transmission of COVID-19 in a general ward setting concluded that: “Vigilance with basic infection control measures, including wearing of surgical masks, hand hygiene and environmental hygiene continues to remain fundamental and essential in the prevention of human-to-human transmissions of SARS-CoV-2”. Unfortunately, during the pandemic, a consistent shortage of PPE has been reported nearly everywhere [6,7]. Professions that have always been considered less exposure-prone may have suffered more from both PPE shortages and minor use experiences than colleagues who already had stored supplies and who were routinely used to wearing PPE and adopting strict infection control procedures [36]. The concept that there are exposure-prone professions may lead some health workers from other specialties to the misconception that they are safe or that they run a low risk of infection. Such a concept has proven to not be useful for airborne diseases such as COVID-19. This may have been the case with GPs in Italy, who seem to have been particularly affected by COVID-19. Stricter basic protocols concerning the use of PPE and cross infection control procedures for all health workers should be introduced, stressed, provided for and demanded.

Chlorhexidine mouthwashes have been frequently adopted in dentistry before starting any procedure, as they have been proven to be effective in reducing patients’ bacterial plaque [37]. During the COVID-19 pandemic, chlorhexidine, povidone-iodine and cetylpyridinium chloride mouthrinses have proven effective in reducing SARS-CoV-2 salivary load [38,39]. Guidelines and recommendations concerning dental care during the COVID-19 pandemic [11,27,28,29,30] suggest to postpone non-essential treatments and/or to reduce aerosol-generating procedures when possible. Not all dental procedures are equally aerosol-producing, and aerosol generation can be controlled, for example, by limiting the use of handpieces and ultrasound and by working with enhanced aspiration devices [40]. A study comparing dental implant treatments during and before the COVID-19 pandemic reported a significant reduction in the use of bone augmentation procedures in 2020, for example [41]. Furthermore, concerning oral surgery, some authors have suggested that dental implant systems requiring minor drilling speed and saline irrigation should be preferred, as they involve less aerosol production [42].

Age is a known risk factor for having more severe forms of COVID-19 [1]. In our analysis, in agreement with the literature, the average age of dead dentists and doctors was between 68 and 70 years. When excluding the population that was over 70, assuming they had retired or limited their activity, the average age was still between 63 and 65 years. Furthermore, all groups (doctors, GPs and dentists) displayed similar ages. Moreover, concerning the geographical distribution of the cases, our results overlap the trend of the corresponding general Italian population, both country-wise as well as region-wise [13].

The COVID-19 pandemic has been a tremendous trigger for the medical community to join efforts aiming to study all possible aspects of this new disease. In recent times, as a result of increasing disease- or vaccine-induced levels of immunity, more effective therapies, improved population awareness on the dynamics of the disease and the presence of a less aggressive variant, restrictions seem to be loosening. Although stronger evidence and indications concerning prevention strategies and treatment options for SARS-CoV-2 infection will continue to build up, the importance of adhering to routine PPE use and infection control procedures should be stressed and should become a prominent part of university education programs. Furthermore, all health professionals should be provided with appropriate PPE and procedure information to safely perform their duty.

## Figures and Tables

**Table 1:** Age distribution of the whole sample, GPs, dentists and remaining doctors.

Birth (Decade)	Whole Sample	GPs	Dentists	Remaining
1910–1919	1	0	0	1
1920–1929	8	3	0	5
1930–1939	27	6	2	19
1940–1949	84	20	6	58
1950–1959	183	96	24	63
1960–1969	32	9	4	19
1970–1979	3	0	1	2
>1980	2	0	0	2
unknown	24	6	1	17
tot	364	140	38	186
average	69 years (range 105–36)	68 years (range 97–55)	68 years (range 89–49)	70 years (range 105–36)

**Table 2:** Age distribution of the whole sample, GPs, dentists and remaining doctors, excluding subjects > 70 years and those with unknown age.

Birth (Decade)	Whole Sample	GPs	Dentists	Remaining
1950–1959	183	94	24	65
1960–1969	32	9	4	19
1970–1979	3	0	1	2
>1980	2	0	0	2
tot	220	103	29	88
average	64 years (range 70–36)	65 years (range 70–55)	65 years (range 70–49)	63 years (range 70–36)

**Table 3:** Geographic distribution of cases.

Geographic Area	Number of Deaths	% of Deaths
Northern Italy (Valle d’Aosta, Piemonte, Liguria, Lombardia, Emilia Romagna, Friuli-Venezia Giulia, Trentino-Alto Adige, Veneto)	215	60%
Central Italy (Lazio, Marche, Toscana, Umbria)	53	14%
Southern Italy (Abruzzo, Basilicata, Calabria, Campania, Molise, Puglia, Sardegna, Sicilia)	96	26%
Total	364	100
Lombardia alone	117	33%

**Table 4:** Standardized Mortality Rates (SMR).

Age Groups	Whole Sample	GPs	Dentists	Remaining
<35	0	0	0	0
35–44	1	0	0	0.300
45–54	0.917	0	0.833	0.571
55–64	1.778	4.644	1.356	0.750
65–74	1.843	5.699	1.566	0.464
75+	1.407	68.669	1.610	0.153

## Data Availability

Data were retrieved by publicly archived datasets whose sources are acknowledged in the “References” section.
